# *Aloe vera* at the frontier of glycobiology and integrative medicine: Health implications of an ancient plant

**DOI:** 10.1177/2050312119875921

**Published:** 2019-09-13

**Authors:** Peter Pressman, Roger Clemens, A Wallace Hayes

**Affiliations:** 1 The Daedalus Foundation, San Clemente, CA, USA; 2 International Center for Regulatory Science, University of Southern California, Los Angeles, CA, USA; 3 College of Public Health, University of South Florida, Tampa, FL, USA

**Keywords:** *Aloe vera*, aloin, anthracenes, glycobiology, polysaccharides, rhein

## Abstract

*Aloe vera* plant extracts are ubiquitous in foods, cosmetics, and medicine. Like all plants, these extracts contain an array of potential bioactives or glycans, which may contribute to health when applied or consumed. In the *Aloe vera* plant, these bioactives are dominated by acemannan, a type of carbohydrate, and related complexes of saccharides, proteins, and lipids. Clinical data suggest aloe extracts may be beneficial in the management of cutaneous and some systemic conditions, such as some forms of immune dysfunction, atherogenesis, malignancy, and numerous cell functions. These extracts also contain an entourage of bioactive substances that may be allergenic and potentially toxic as well as salutary. These substances include aloin and a variety of anthracenes. The concentrations of potential allergens, aloin, and related compounds are markedly reduced through controlled decolorization processes that are utilized by leading Aloe products manufacturers. The entourage effects of contemporary *Aloe vera* when consumed or applied topically represent opportunities for clinical investigation which may be applied to commercial consumer products and therapeutic indications. Future research should fully explore the range of bioactive glycan components and their respective safety and efficacy. The history and ongoing popularity of *Aloe vera* products represent a pragmatic mandate for well-designed investigation into the diverse functional roles of glycans.

## Introduction

**Figure 1.**
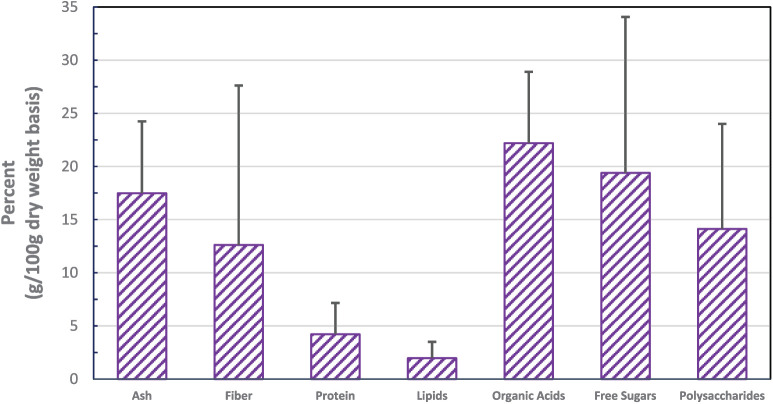
Major components of *Aloe vera* (mean ± SD; n = 19). Source: Adapted from Zhang et al.^1^

*Aloe vera* is a succulent species of the genus Aloe belonging to the Asphodelaceae (Liliaceae) family, and it appears shrubby and pea-green in color. Aloes are evergreen perennials that likely originated from the Arabian Peninsula, but grow wild in tropical climates globally and are cultivated for agricultural, cosmetic, and medicinal uses. The *Aloe vera* plant is the source of *Aloe vera* gel (AVG), which is obtained from the interior of the fleshy leaves of the *Aloe vera* plant. A “latex” or the aloin component is found in the pericarpal layer just below the rind of the fleshy leaves. Despite tremendous differences in cultivars, geographic region, season at harvest, agricultural practices, and specific processing methods, the general classes of intrinsic compounds of this plant can be summarized, albeit with significant variability in percentage by dry weight (Figure 1).

Aloes can adapt to habitats with low or erratic water availability, are characterized by the capacity to store significant volumes of water in their tissue, and are able to use crassulacean acid metabolism, an adaptation to the photosynthetic pathway that involves the formation of malic acid.^2^

Use of AVG extracts in foods and beverages and in moisturizing cosmetics and soothing topicals seemed to blossom from a long-standing agricultural milieu and relatively small cottage industry to a major enterprise during the 1970s.^3^ *Aloe vera* preparations have been used topically with the intent to promote healing of wounds and for the management of various cutaneous conditions and orally as a laxative.^4^ It is the dried latex of some Aloe species, such as *Aloe ferox* Miller (Cape aloe or bitter aloe), that has been used as a laxative.^5^

Presently, *Aloe vera* is also marketed and used as a remedy for a variety of systemic conditions and is found increasingly in dietary supplements and food products. AVG is readily found in hundreds of skin products, including lotions and sun blocks.^6^

Juice from the plant is obtained by grinding or macerating the entire *Aloe vera* leaf followed by purification to remove the undesirable phenolic compounds found in the latex. This purification step is usually accomplished via activated carbon filtration in a process known as decolorization.

*Aloe vera* juice can also be obtained by stripping away the outer leaf rind, rinsing or washing away the latex, and processing the remaining inner leaf material. Decolorization is also typically employed with this method.

Aloe plants have in common green fleshy leaves covered by a thick cuticle or rind and an inner clear pulp. The rind, which constitutes approximately 20% to 30% by weight of the entire plant leaf, lends turgidity to the leaf. Vascular bundles are located in the leaf pulp just beneath the rind and contain the impressive volumes of aqueous juice.

The latex or aloin component contains many bioactive components, including potentially toxic levels of the mutagenic anthraquinones, which are also responsible for the strong laxative effects of the latex. The latex also contains rhein (4, 5-dihydroxyanthraquinone-2-carboxylic acid), a lipophilic anthraquinone which is found in a variety of medicinal herbs that have been used in traditional Chinese medicine (TCM) for more than 1000 years. Emerging evidence suggests that rhein has potential salutary pharmacological effects, including hepatoprotective, nephroprotective, anti-inflammatory, antioxidant, anticancer, and antimicrobial activities.^7^ The dried form of the latex, termed “aloe,” is a “drug” which is regulated by the Food and Drug Administration (FDA). The leaf pulp of the plant is composed of large thin-walled parenchymal cells that contain the clear mucilaginous aqueous extract called AVG. While the aloe latex (“Aloe sap”) is derived from the total leaf, the “*Aloe vera* gel” components are derived from the leaf pulp and are not the same.

It is important to note that some “Aloe” products utilize the total ground leaf and will therefore contain all the above ingredients, possibly compounded with other botanical or herbal extracts. A casual Internet search demonstrates myriad corporate entities that market all manner of Aloe in the absence of a regulatory framework or identity standards. There are, however, a handful of large and long-standing Aloe companies that control all aspects of production from agriculture to processing to marketing and distribution, and these few strive to meet US and international standards.

## Cultivation and production of *Aloe vera* extract

The succulent leaves are handpicked at maturity, the plant then being at least 1 m in height and approximately 3 years old. The fleshy leaves are then quickly taken to the extraction facility to mitigate contamination and enzyme oxidization of the leaf gel. The ultimate safety of products containing AVG resides in the production process and quality assurance measures.

The typical approach to production involves washing the freshly harvested *Aloe vera* leaves in a bactericide, followed by processing of the leaves to mechanically separate the gel matrix from the outer cortex. *Aloe vera* products are generally produced by cold extraction of the leaf, which is called a filet. Either the whole leaf or the soft, gelatinous filling may be used. In the early to mid-1980s, a process called the “whole leaf” process was developed and is now used by many Aloe producers. This method involves grinding up the whole leaf, including the rind, and then filtering and carbon-treating the resulting slurry to produce “whole leaf *Aloe vera*,” a clear and brackish tasting product that resembles water.^8^

As noted above, AVG is obtained from the *Aloe vera* leaf pulp. The outer layer of the leaf contains most of the aloin (anthraquinone), which is responsible for the irritating purgative or laxative effect of aloe as well as its toxic effects at higher levels of exposure. Therefore, the food and dietary supplement producers of *Aloe vera* typically remove the outer layer from products intended for ingestion. The mechanical extrusion of the mucilaginous gel from the fibrous fraction of the pulp gives a 70% yield with a water content of 99%–99.5%. The gel of field-grown *Aloe vera* is reported to have a pH of 4.4–4.7 and a total soluble solids content of 0.56%–0.66%. The high acidity of the AVG is likely due to the accumulation of organic acids, such as malic acid, in the cells of the pulp.^9^

Alternatively, the aloe gel can be exposed to a heat treatment process. In this thermal process, sterilization is achieved by subjecting the aloe liquid obtained from the activated carbon treatment to pasteurization at high temperature. The biological activity of AVG appears to remain intact when the gel is heated at 65°C for periods of less than 15 min.^10^

## Glycobiology

The emerging field of glycobiology is the study of different forms of saccharides or carbohydrates of plant origin, that is, the polymannan polysaccharides of Aloe. In particular, the focus is on their biosynthetic activity that may offer an innovative nutritional approach to a range of pathologies.^11^

Intrinsic to glycobiology is glycosylation. This process is the most common form of protein and lipid modification, where saccharides are attached to proteins and lipids through a complex, but ordered, process in the ribosome, endoplasmic reticulum, and Golgi bodies of the cell to enable intracellular functioning and cell-to-cell communication.^12^ It cannot be over-emphasized that glycoconjugates, glycolipids, glycoproteins, and proteoglycans, are critical components of the cell surface recognition process throughout all organ systems.^13^

Varki^14^ has asserted that with respect to structural diversity, glycans have the capacity to far exceed proteins and nucleic acids. Their structural diversity allows them to encode information for specific molecular recognition and to serve as determinants of protein folding, stability, and pharmacokinetics. In light of the fact that glycosylation is one of the most common forms of posttranslational modification, increasing numbers of investigators are undertaking research in glycobiology and glycosylation; the attachment of saccharide units to proteins and lipids.

Despite detailed characterization of the components that carry out glycosylation, a complete picture of a cell’s glycoconjugates remains elusive because of the experimental challenges inherent in characterizing complex carbohydrates in cellular compartments, extracellular spaces, and body fluids. Elegant single-molecule force spectroscopy (SMFS) by atomic force microscopy (AFM) has enabled the molecular interactions of sugars to be studied, and single-molecule fluorescence microscopy and spectroscopy have led to a preliminary understanding of the role of non-sweetening sugars in biological processes, which have in turn suggested interactions between saccharides and their transporters in in vitro systems.

Glycobiology has even driven techniques for the induction of adult stem cells via exposure to complex carbohydrates of plant origin.^15^ Historically, it has been pointed out that dietary supplementation with high dose oligosaccharides has demonstrated the potential for benefit in the following: cancer,^16^ HIV/AIDS,^17^ immune system functioning,^18^ hyperlipidemia^19,20^ atherogenesis,^21^ chronic fatigue syndrome,^22^ and attention deficit hyperactivity disorder.^23^

## *Aloe vera* and glycans

AVG is a water-gel system that contains the putative bioactive glycan, acemannan, plus amino acids, other carbohydrates, organic acids, and vitamins. Specifically, the gel is colorless and contains <2% solids, of which about 78% of the solids are acemannan. Specifically, acemannan is a long-chain poly-dispersed beta-(1,4)-acetylated poly-mannose with interspersed acetyl groups, with a mannose monomer/acetyl ratio of approximately 1:1. Again, it is important to underscore the point that the health properties of topically applied and orally consumed *Aloe vera* are usually attributed to this glucomannan known as acemannan.^24^ The presence of the medium to higher molecular weight acetylated polymannan polysaccharides is viewed as a proxy for the biological activity found in *Aloe vera*.

Apart from the spectrum of polysaccharides and anthraquinones, *Aloe vera* contains at least 75 other potentially active constituents: vitamins, enzymes, minerals, sugars, lignin, saponins, salicylic acids, and amino acids.^1^ The accumulated experimental work to date together with albeit limited clinical study has suggested a daunting array of glycobiological functions that independently and in intriguing fashion parallel emerging applications of *Aloe vera*. These functions may include but are not limited to the following: physical protection and tissue elasticity, lubrication, physical expulsion of pathogens, diffusion barriers, protection from proteases, cell migration and wound healing, modulation of membrane receptor signaling, depot (hydrophilic) functions, protection from immune recognition, epigenetic histone modifications, prebiotic support, antigen recognition, uptake and processing, and intercellular signaling. This impressive list of claims is discussed in detail in the following section on clinical research.

## Clinical studies

Confusion among consumers and health care providers exists with regard to the form of Aloe, its matrix, route of administration (topical versus oral), and the specific indications for a given use. Highly variable clinical research quality also appears to have obfuscated the health claims picture. Small case series, in vitro work, animal models, ecological correlational studies, and individual testimonials of benefit or lack thereof are insufficient to adequately demonstrate safety or efficacy. The distinction between statistically significant results and clinically meaningful effects seems generally lacking. Nevertheless, selected recent work is provocative and suggestive of genuine benefit. It is still fair to say that the clinical use of *Aloe vera* is supported mostly by persuasive anecdotal data.

One intriguing study found that fructans from the plant induced bacterial growth better than inulin (commercial fructo-oligosaccharide). The study concluded *Aloe vera* extract has shown evidence of prebiotic potential. In addition, it was reported that aloe appeared to reduce gastrointestinal distress by increasing pepsin production and mucus secretion and decreasing HCl acid production.^25^

Another 4-week study among 44 patients with inflammatory bowel disease (IBD) reported some remission or improvement during the *Aloe vera* intervention period, thereby indicating a therapeutic potential when administered at 100 mL twice a day.^26^ The threat to validity here is the failure to identify the probiotic strain and the specific (prebiotic) carbon source required for its metabolism and ultimate production of compounds that may promote better health.

A 2015 study through the Royal Society of Chemistry also concluded that incorporating *Aloe vera* into food products can play a beneficial role in prebiotic health.^27^ Overall, the plant has been shown to exhibit gastric anti-secretory activity and could protect the gastric mucosa at lower concentration against injurious agents. It is certainly not unreasonable to speculate that the glycans in Aloe serve as prebiotic fuel for probiotic organisms as well as gut commensals.

The gels of *Aloe vera* contain immunomodulatory components, demonstrated in vitro, including aloctin A and acemannan. Some work has suggested that AVG increases ovalbumin-specific cytotoxic T lymphocyte generation and suppresses bacterial-induced pro-inflammatory cytokines from human immune cells in those with diabetes.^28^

Metabolic syndrome has traditionally been viewed as a constellation of conditions that elevate the risk of heart disease, stroke, and diabetes. A 2012 double-blind study examined the effects of *Aloe vera* supplementation in human subjects with prediabetes/metabolic syndrome. The results showed standardized *Aloe vera* preparations offered a potential strategy to attenuate the impaired fasting glucose and impaired glucose tolerance observed in conditions of prediabetes/metabolic syndrome.^29^

A clinical study of 5000 patients treated with AVG observed reduction in total serum cholesterol, serum triglycerides, fasting and post-prandial blood sugar level in diabetic patients, total lipids, and also an increase in high-density lipoprotein (HDL).^30^

An early systematic review utilized independent literature searches conducted in MEDLINE, EMBASE, Biosis, and the Cochrane Library.^31^ Only controlled clinical trials (on any indication) were included. Analysis suggested that oral administration of *Aloe vera* might be a useful adjunct for lowering blood glucose in diabetic patients as well as for reducing serum lipid levels in patients with hyperlipidemia.

In the same study, topical Aloe was viewed as possibly effective for reducing intensity and duration of genital herpes infection and flares of psoriasis. Promotion of wound healing was unclear. The authors concluded that even though there are some broad promising results, clinical effectiveness of oral or topical administration was insufficient to support firm conclusions or recommendations relative to the identified outcomes.

A Pacific Rim group recently reported that AVG offered no protective effects against damage secondary to radiotherapy-induced dermatitis.^32^ More emphatic and positive findings were reported in a prospective, albeit in vitro, study that explored aspects of wound healing.^33^ These authors reported data indicating that both AVG and Cape Aloe extract (CAE) significantly improved wound healing in human primary epidermal keratinocytes (HPEKs) and a human skin equivalent model. In addition, flow cytometry analysis revealed that cell surface expressions of β1-, α6-, β4-integrin, and E-cadherin increased in HPEKs treated with AVG and CAE. These increases were hypothesized to contribute to cell migration and wound healing. Treatment with *Aloe* also resulted in significant changes in cell-cycle progression and in increases in cell number. *Aloe* was associated with increased gene expression of differentiation markers in HPEKs, suggesting roles for AVG and CAE in the improvement of keratinocyte function. Furthermore, human skin epidermal equivalents developed from HPEKs with medium containing *Aloe* were thicker than control equivalents, suggesting the effectiveness of *Aloe* on enhancing epidermal development.

A recent Australian study noted that despite its widespread use as a cost-effective agent for wound management, there is minimal clinical evidence on the efficacy of *Aloe vera* in wound healing.^34^ The investigator undertook to identify evidence focusing on efficacy of *Aloe vera* for healing partial thickness burns, diabetic ulcers, leg ulcers, surgical wounds, biopsy sites, and pressure injuries. The study findings were contradictory, however, since *Aloe vera* was not found inferior to other contemporary wound care products, particularly for burn management.

In contrast, more dramatic findings were reported in a study comparing and evaluating efficacy of *Aloe vera* mouthwash on clinical levels of dental plaque in a general population with established antiplaque agent of 0.2% chlorhexidine gluconate and placebo using the approach of a 4-day plaque regrowth protocol.^35^ *Aloe vera* mouth wash was found to have an efficacy comparable to the antiplaque agent 0.2% chlorhexidine gluconate mouthwash. The authors asserted that considering the side effects associated with chlorhexidine, *Aloe vera* mouthwash might be considered as a viable alternative.

Another group looked at the efficacy of an *Aloe vera* mouthwash compared with a benzylamine mouthwash in the management of radiation mucositis in head and neck cancer patients using a triple-blind, randomized controlled trial.^36^ The authors found that *Aloe vera* mouthwash was as beneficial as benzylamine mouthwash in reducing the severity of radiation mucositis and presented no adverse reactions.

An Indian group found that adjunctive use of locally delivered AVG, in comparison to locally delivered placebo gel, was associated with greater reduction in plaque index, modified sulcus bleeding index, and probing depth as well as more gain in clinical attachment level in patients with Type 2 diabetes mellitus and chronic periodontitis.^37^

In an interesting in vitro study, a cytotoxicity assay showed that *Aloe vera* in prearranged concentrations was cell-compatible and indicated a dose-dependent antiviral effect of *Aloe vera*.^38^ The inhibitory effect of various concentrations of *Aloe vera* was observed 1 h after the Vero cell was infected with herpes simplex virus 1 (HSV-1). The findings showed a significant inhibitory effect of 0.2%–5% AVG on the HSV-1 growth in the Vero cell line.

A group of orthopedic surgeons reported a randomized, triple-blinded clinical trial that was done on 80 in-patients in an orthopedic ward in Iran in 2016. In light of observations that AVG appeared to reduce expected increase in temperature, non-blanchable redness, swelling, and pain of the skin of regions under study, applying it toward reducing the likelihood of development of grade I pressure ulcers in at risk patients was recommended.^39^ No follow-up has been published as of this writing.

A team of plastic and reconstructive surgeons reported that topical AVG demonstrated significant split-thickness skin graft donor site healing, but failed to provide pain relief. Perioperative nutritional status, age, co-morbid conditions, and medications were not investigated as potential confounds.^40^

## Toxicology

Although there are, at best, only limited epidemiological data available regarding the toxicity and/or carcinogenicity of *Aloe vera* (*Aloe barbadensis* Miller), adverse effects in rodents have raised questions of its potential toxicity in humans.^41^ To confound the issue, most toxicity and safety assessment studies in animals have been conducted on the non-decolorized whole leaf extract (WLE) which contains a number of Aloe latex compounds, such as phenolics that include anthraquinone *C*-glycosides, anthrones, and free anthraquinones.^2^ Furthermore, most of the studies dealing with the toxicity and safety of *Aloe vera* review here were difficult to interpret because the specific aloe extract was not identified or fully characterized, or the content and/or concentration of the potential toxins, such as hydroxyanthracene glycosides, especially emodin or aloin A (barbaloin), were not reported.

Some studies have suggested that *Aloe vera* exposure causes toxicity while other studies have reported no untoward effects. For example, the hydroxyanthracene glycosides in *Aloe vera* have been reported to be genotoxic to bacteria and mammalian cells. Among other untoward effects of these glycosides in extracts of *Aloe vera* are their interactions with ultraviolet (UV) radiation leading to selected exon mutations in the p53 tumor-suppressor gene in a mouse model.^42^ The investigators speculated that the spectrum and distribution of mutations induced by the anthraquinone emodin may be relevant to human skin health.

The parenteral LD_50_ of AVG has been reported to be >200 mg/kg in mice, >50 mg/kg among rats, and >50 mg/kg in dogs. The respective intravenous studies resulted in an LD_50_ of >80 mg/kg using mice, >15 mg/kg among rats, and >10 mg/kg in dogs.^43^

A 2013 report indicated that decolorized *Aloe vera* juice did not increase mutagenesis using the *Salmonella typhimurium* TA100 strain.^44^ The DNA damage repair assay demonstrated the absence of dose-related increase in SOS transgene induction. In addition, the juice did not induce DNA damage repair in the presence of S9 extract at levels up to 5×; however, at 10×, the juice appeared to be either cytotoxic or bacteriostatic in the *Escherichia coli* system. The investigators concluded that the effect on bacterial growth was not indicative of increased DNA damage. To further understand these results, it is important to note that S9 is a rat liver extract treated with a carcinogen intended to induce enzyme systems associated with procarcinogen metabolism. It is also noteworthy that the SOS response is a global response to DNA damage in which the normal cell cycle is arrested and that DNA repair with mutagenesis is induced. This process varies among species, and the results to DNA changes are inconsistent.

In a frequently cited toxicology study of commercial decolorized *Aloe vera* juice, whole leaf *Aloe vera* was treated with activated charcoal to remove the latex portion of the plant and studied for genotoxicity and mammalian toxicity.^45^ The processed juice was reported non-genotoxic both in histidine reversion (Ames Assay) and DNA repair assays. When fed to male and female F344 rats over 13 weeks, decolorized whole leaf (DCWL) aloe produced no toxicity as assessed by behavior, stools, weight gain, feed consumption, organ weights, and hematologic or clinical chemistry profiles. These results were diametrically opposed to those obtained using preparations containing aloe latex phenolic compounds such as anthraquinones reported in the study sponsored by the National Toxicology Program.

In Guo et al.’s^46^ study, an *Aloe vera* WLE, which contains more than 200 unique substances, and a decolorized whole leaf extract (WLD) were studied for potential toxicity and carcinogenicity. Both extracts showed cytotoxic and genotoxic effects, but at different concentration ranges (0–8 mg/mL) using the mouse lymphoma assay (MLA). WLE was clastogenic at 4.0 mg/mL with roughly 20% cytotoxicity. In the case of WLD, cytotoxicity reached approximately 70% before the decolorized extract was clastogenic, suggesting some of the clastogenic/cytotoxic components of the whole leaf extract were removed by the activated carbon filtration. The investigators concluded that while the decolorization process removed approximately 99% of the anthraquinones, a proxy for the vast array of the aloe latex substances, some mutagenic characteristics, remained in the extract based on the MLA.

Another approach to assessing safety of AVG involved supercritical CO_2_ extraction.^47^ The AVG was negative in the bacterial reverse mutation test (Ames assay) using *S. typhimurium* strains (TA98, TA100, TA1535, TA 1537) and in *E. coli* (Wp2uvrA) with and without metabolic activation up to 5000 µg/plate. The extract was also negative in a chromosomal aberration test in Chinese hamster lung cells at 1600 µg/plate and in an in vivo bone marrow micronucleus test at 150 mg/kg/day. The supercritical CO_2_ extract was given orally by gavage to male and female Crl:CD (SD) rats for an acute, single-dose study at 150 mg/kg and daily for 90 days at concentrations of 0, 30, and 150 mg/kg. Animals in the single-dose oral toxicity study did not present any abnormal pathologies of any examined organ relative to the control. Within the 90-day study, none of the groups differed in food consumption or body weight change. Other than a few variations of some hematological assessments relative to controls, the values were within normal ranges regardless of study group assignment. In the histopathology within the 150 mg/kg group, male animals presented moderate atrophy of reproductive structures. Slight eosinophilic infiltration of kidney tissues was observed in male and female animals at this higher dose. Importantly, this *Aloe vera* extract was non-mutagenic based on the Ames test, the chromosomal aberration test, and in an in vivo bone marrow micronucleus test. Animals in the acute and 90-day subchronic toxicological assessments did not present any adverse effects.

The German Federal Institute for Risk Assessment (BfR) assessed the possible health risks of *Aloe vera* food supplements. BfR stated that the outer layers of the leaves of *Aloe arborescens* are of toxicological relevance. As with all more than 400 Aloe species, these layers contain plant-based anthranoids, which have long been suspected of having a genotoxic and carcinogenic effect.^48^ In addition to data on the pure substances isolated from these species, the Germans have conducted tests on anthranoid-containing preparations made from Aloe leaves. The results of their long-term studies confirm the suspicion of carcinogenicity; however, the investigators admit that there are data gaps which should be closed with regard to the details and mechanisms of cancer development.

## Limitations of the present review

We did not address the entire scope of medical and pharmacologic claims involving *Aloe vera*. First, the absence of standardization in agricultural and processing variables threatens the validity, reliability, and generalizability of research that aims to evaluate mechanisms and efficacy. In addition, the significantly uneven character of the existing research itself presents a daunting challenge. The vast majority of the world literature is case report or historical narrative in the traditional medicine, natural medicine, or complementary and alternative medicine space. In the more rigorous scientific realm, in vitro work further complicates achieving a coherent and systematic critical picture of the clinical field. Follow-on work, in the style of a Cochrane review, may be possible in the near term given the apparent excitement and expansion of *Aloe vera* research.

## Conclusion

In the increasingly dynamic discussion at the interface of plant-based foods, glycobiology, and health, *Aloe vera* is unique. It is ubiquitous in cosmetics, foods, and dietary supplements. Despite Aloe’s rich history and vast applications, the chemistry and biological applications remain uncertain. Modern decolorization processing methods used by legitimate manufacturers allow efficient removal of toxic aloin and anthracene constituents, and these methods herald the potential development of a spectrum of novel food and cosmetic products that may have clinically significant health effects. These salutary health effects more than likely can be attributed to putative bioactive glycan components in *Aloe vera* which may behave individually or in an entourage manner; however, while highly likely, the safety and superiority to standard oral and transdermal interventions for a variety of health problems have yet to be shown unequivocally. Dry skin, sunburn, wound healing, dental, and oral hygiene are among the most common topical applications. Side effects have been reported in the literature, but the reported adverse effects have been attributed to forms of the AVG that are almost exclusively from products containing non-decolorized *Aloe vera* which may contain an array of toxins innate to the plant, but which can be removed by modern decolorization processing methods.

Systemically consumed Aloe is also claimed as effective in a variety of applications from weight management to various malignancies. A principal challenge in making sense of the health claims is that the current medical and scientific literature is highly variable and often divided in character and quality, often utilizing the toxic non-decolorized gel, in vitro and animal models, poorly designed clinical series, and a wide variety of topical and systemically administered forms.

Combined with the accelerating power of genomics and glycomics, the field has now reached a point where the numerous biological roles of glycans have been elucidated at least to the extent that research and development efforts appear emphatically justified.

The real excitement and therapeutic potential of *Aloe vera* products lie at this point with the hitherto under explored potentials of the glycans, the polymannan polysaccharides, and lipid soluble non-carcinogenic anthraquinone compounds such as rhein.

The goals of ongoing and future research should focus on specific cultivars of Aloe processed in a standardized manner, with meticulous elucidation of the full range of active components pre- and post-extraction, along with comprehensive safety (toxicologic) profiling for specific indications via specific routes of administration. The ancient *Aloe vera* plant may yet hold surprises that drive valuable lines of medical research and product development.
